# Transcriptomic analysis at organ and time scale reveals gene regulatory networks controlling the sulfate starvation response of *Solanum lycopersicum*

**DOI:** 10.1186/s12870-020-02590-2

**Published:** 2020-08-24

**Authors:** Javier Canales, Felipe Uribe, Carlos Henríquez-Valencia, Carlos Lovazzano, Joaquín Medina, Elena A. Vidal

**Affiliations:** 1 Instituto de Bioquímica y Microbiología, Facultad de Ciencias, Universidad Austral de Chile, Valdivia, Chile; 2 Millennium Institute for Integrative Biology (iBio), Santiago, Chile; 3 Centro de Biotecnología y Genómica de Plantas (CBGP), Instituto Nacional de Investigación y Tecnología Agraria y Alimentaria (INIA), Universidad Politécnica de Madrid (UPM), Madrid, Spain; 4 Centro de Genómica y Bioinformática, Facultad de Ciencias, Universidad Mayor, Santiago, Chile; 5 Escuela de Biotecnología, Facultad de Ciencias, Universidad Mayor, Santiago, Chile

**Keywords:** Sulfate, Transcriptomics, Gene networks, Transcription factors, Starvation, Roots, Leaves, Tomato, *Solanum lycopersicum*, SLIM1

## Abstract

**Background:**

Sulfur is a major component of biological molecules and thus an essential element for plants. Deficiency of sulfate, the main source of sulfur in soils, negatively influences plant growth and crop yield. The effect of sulfate deficiency on plants has been well characterized at the physiological, transcriptomic and metabolomic levels in *Arabidopsis thaliana* and a limited number of crop plants. However, we still lack a thorough understanding of the molecular mechanisms and regulatory networks underlying sulfate deficiency in most plants. In this work we analyzed the impact of sulfate starvation on the transcriptome of tomato plants to identify regulatory networks and key transcriptional regulators at a temporal and organ scale.

**Results:**

Sulfate starvation reduces the growth of roots and leaves which is accompanied by major changes in the organ transcriptome, with the response being temporally earlier in roots than leaves. Comparative analysis showed that a major part of the Arabidopsis and tomato transcriptomic response to sulfate starvation is conserved between these plants and allowed for the identification of processes specifically regulated in tomato at the transcript level, including the control of internal phosphate levels. Integrative gene network analysis uncovered key transcription factors controlling the temporal expression of genes involved in sulfate assimilation, as well as cell cycle, cell division and photosynthesis during sulfate starvation in tomato roots and leaves. Interestingly, one of these transcription factors presents a high identity with SULFUR LIMITATION1, a central component of the sulfate starvation response in Arabidopsis.

**Conclusions:**

Together, our results provide the first comprehensive catalog of sulfate-responsive genes in tomato, as well as novel regulatory targets for future functional analyses in tomato and other crops.

## Background

Sulfur (S) is an essential nutrient for living organisms, as main component of important proteinogenic amino acids methionine (Met) and cysteine (Cys), and of numerous coenzymes and prostethic groups such as iron-sulfur centers, S-adenosylmethionine (SAM), glutathione and coenzyme-A, among others [1, 2]. As such, S is directly involved in a wide variety of metabolic processes in plants, including photosynthesis or nitrate reduction and assimilation [2]. S is also a component of the thioglucosides glucosinolates (GSLs) in *Brassicales* and allyl Cys sulfoxides in *Allium* species, important secondary metabolites involved in defense responses [1, 3]. Importantly, plants are a main source of reduced S for animals and humans, who are unable to assimilate inorganic S and must obtain it from organic compounds such as proteins in their diet. As a macronutrient, the average concentration of S in plant tissues for adequate growth is relatively high (around 0.1 to 0.5% of plant dry weight) [4]. However, S deficiency has become a rising problem in crops primarily due to decreases in atmospheric S inputs caused by strict industrial emission regulations and changes in agronomic practices, including the use of fertilizers with reduced S compositions, reduction in the use of S-containing fungicides and intensive cropping systems that remove S from the soils [5]. Prolonged S deficiency causes a reduction in photosynthetic rate, chlorophyll levels and amino acid contents, leading to growth retardation and diminished yield, nutritional quality and taste of crops [5–7]. Moreover, S-starvation alters the acquisition of other nutrients such as nitrate, phosphate, molybdenum, selenium, and iron [8–11]. In this framework, understanding the regulation of the S-starvation response and how it impacts the acquisition and metabolism of S, as well as other processes in plants has become a major interest for research and crop improvement.

Sulfate is the most stable form of S in soils, and thus the main source of S for plants [1]. Sulfate is taken up by root epidermal cells via the action of high affinity sulfate transporters (SULTRs) [12], mainly SULTR1;1 and SULTR1;2 in Arabidopsis [13, 14]. Sulfate can then be transported to the aerial tissues by specific transporters such as SULTR2;1 and SULTR3;5 or stored in vacuoles through the action of SULTR4;1 and SULTR4;2 [12]{Citation}. The first step in sulfate assimilation is catalyzed by ATP sulfurylase (ATPS), generating adenosine 5′-phosphosulfate (APS). At this point, APS can be reduced by APS reductase (APR) and sulfite reductase to sulfide, which is incorporated into O-acetylserine (OAS) to form cysteine, or can be phosphorylated by APS kinase to 3′-phosphoadenosine 5′-phosphosulfate (PAPS) [1, 2]. PAPS, can be utilized as a sulfate donor to synthesize a variety of sulfated metabolites such as GSLs, brassinosteroids, sulfoflavonoids, phytosulfokines and sulfojasmonates [3].

Sulfate deficiency or starvation triggers important changes in sulfate acquisition and metabolism in plants, such as the catabolism of S-storage compounds and repression of processes involved in the biosynthesis of secondary S metabolites [15]. These changes involve upregulation of sulfate transporters, accumulation of OAS and degradation of GSLs [15]. This complex rearrangement of plant metabolism is partly explained by changes that occur at the transcriptional and post-transcriptional levels. At the transcriptional level, S-responsive elements (SUREs) sequences have been identified in the promoter regions of *SULTR* genes, *APR* genes and of other sulfate deficiency-controlled genes [16–19]. In the last years, multiple efforts have been carried out to identify the regulatory factors that control the expression of sulfate starvation-responsive genes. Using genetic approaches, different transcription factors (TFs) have been shown to be involved in sulfate transport and/or assimilation control, including MYB family members, LONG HYPOCOTYL 5 (HY5), and the EIL family TF SULFUR LIMITATION 1 (SLIM1) [20–23]. SLIM1 has arisen as an important TF controlling the sulfate deficiency response [24]. This TF is a transcriptional regulator of sulfate transporters and GSL synthesis genes, among other sulfate-deficiency controlled genes in Arabidopsis [23, 24]. At the post-transcriptional level, microRNA395 (miR395) targets the low-affinity transporter SULTR2;1 and three members of the ATPS gene family [25]. miR395 is highly induced by sulfate starvation and acts downstream SLIM1 to control sulfate assimilation and homeostasis [26–29]. Moreover, the nuclear protein MORE SULPHUR ACCUMULATION 1 (MSA1) has been shown to control SAM biosynthesis, affecting global DNA methylation and causing the hypomethylation of the *SULTR1;1* and *SULTR1;2* transporter genes and the *APR3* gene, indicating a possible epigenetic mechanism of control of S homeostasis in the plant [30].

The utilization of -omics approaches to study plant responses to S availability in the last decades has significantly advanced our understanding of the molecular mechanisms and physiological processes regulating S metabolism and homeostasis in plants [1, 31]. A myriad of genes differentially expressed in response to sulfate deficiency have been identified using transcriptomic approaches, mostly in Arabidopsis [10, 16, 17, 23, 32–38] and *Triticum aestivum* [39–42]. Meta-analysis of transcriptomics data has shown that sulfate deficiency-responsive genes are enriched in biological processes related to sulfate transport and metabolism, but also in processes related to cell wall organization, regulation of proteolysis, and metabolism of carbon and nitrogen [43]. The sulfate deficiency response has also been explored by metabolomics and proteomics analyses in Arabidopsis and crops [10, 17, 32, 35, 44–52]. Furthermore, systems biology approaches that integrate multi-omics data have been adopted to generate a holistic vision of the sulfate-deficiency response [31]. These integrative approaches have allowed for the identification of TF candidates involved in the sulfate-deficiency response such as MYB28 and MYB29 [44, 53], IAA28 [54, 55], NF-YA2 and RVE2 [43], among others [17].

Tomato (*Solanum lycopersicum*) is one of the most important vegetable crops in the world [56]. In 2018, the total world area harvested for tomatoes was 4.7 million ha, with an annual production quantity of 182 million tons (http://fao.org/faostat/en/#data/QC). Tomato produces fleshy fruits important for the human diet as a source of vitamins and carotenes such as beta-carotene and lycopene [57]. Sulfate deficiency severely reduces the growth of tomato plants [58–62], diminishing biomass, protein concentration, total S in shoots and roots [61, 62], chlorophyll contents [59–61], as well as yield [63]. Given the economic importance of tomato worldwide, understanding how tomato responds to sulfate starvation at the molecular level and identifying key regulatory components is of paramount importance for sustainable tomato production in the present and future agricultural scenarios.

In this work, we identified genes and biological processes that participate in the sulfate starvation response of tomato, at a temporal and organ scale using transcriptomics and network analysis. Integration of expression and regulatory TF-target interaction data allowed us to pinpoint key candidate TFs that might coordinate the temporal growth response of roots and leaves to external sulfate availability.

## Results

### Sulfate starvation has a major impact on leaf and root growth in early stages of tomato development

**Fig. 1:**
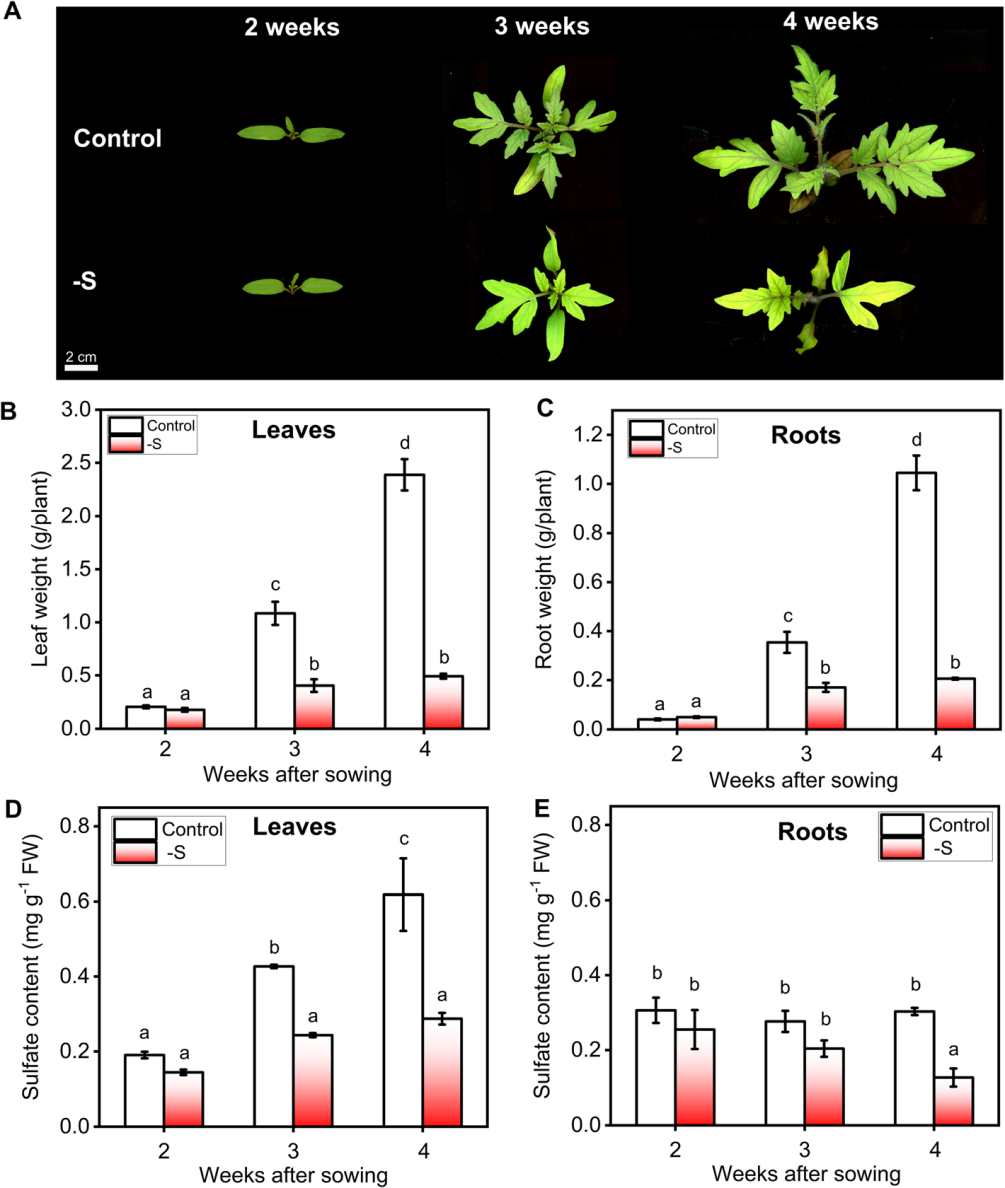
External sulfate availability is required for optimal plant growth. **a** Representative images of tomato plants (*Solanum lycopersicum* cv. Moneymaker) grown under full nutrient or S-limiting conditions for 4 weeks. Biomass accumulation is reduced by sulfate deficiency from 3 weeks after sowing. Fresh weight for leaves (**b**) and roots (**c**) were measured from 2 to 4 weeks after sowing. Sulfate content of leaves (**d**) and roots (**e**) were determined from 2 to 4 weeks after sowing using the turbidimetric method [27]. Values plotted correspond to the means of three independent experiments ± the standard error of the mean. Means with different letters indicate significant differences (*P* < 0.05 two-way ANOVA and Tukey’s test). 3–5 different plants were measured for each experimental replicate

As previously reported for other tomato cultivars [58–63], we found that the growth of *Solanum lycopersicum* cv. Moneymaker seedlings was severely impacted by sulfate starvation. As shown in Fig. 1a, lack of external sulfate has a clear negative effect on the growth of the aerial tissue, as compared with the full nutrient condition. This effect is noticeable from the third week after sowing. Multifactorial analysis of leaf fresh weight and total leaf area showed that there is a strong interaction between sulfate availability and time factors (*p*-value < 0.001, two-way ANOVA, Fig. 1b and Figure S1), indicating that the growth response of leaves to sulfate availability depends on the plant developmental stage. Accordingly, no significant differences between control and sulfate-starved plants were observed at 2 weeks after sowing (*p*-value = 0.81), while a strong dependence of external sulfate on leaf fresh weight and area was observed 3 and 4 weeks after sowing (*p*-value < 0.001) (Fig. 1b and Figure S1). The same interaction between sulfate and time was observed for root weight, with differences between control and sulfate-starved plants being significant only after 3 and 4 weeks of sowing (*p*-value < 0.001, Fig. 1c). These results indicate that whole-plant growth is severely affected by the lack of external sulfate from 3 weeks after sowing.

Consistent with leaf weight and leaf area measurements over time, there is a steady accumulation of sulfate in leaves, that is abolished in sulfate-starved plants in weeks 3 and 4 (Fig. 1d). In contrast, the increase in root weight over time of plants grown in control condition is not explained by a concurrent increase in root sulfate levels (Fig. 1e). Moreover, no major differences in sulfate accumulation are observed in the roots of full nutrient or sulfate-starved plants (Fig. 1e). Together, these results indicate that additional factors besides sulfate content might be controlling root growth response to sulfate starvation.

### Sulfate starvation promotes major changes in leaf and root transcriptomes in a time-dependent manner

In order to gain insights into the molecular mechanisms underlying leaf and root growth repression in response to sulfate starvation, we performed a transcriptomic analysis at a temporal and organ scale, using RNA-seq. To perform this analysis, total RNA from roots and leaves was extracted at 2, 3 and 4 weeks after sowing, considering three independent biological replicates. The RNA-Seq data was pseudo-aligned to the ITAG3.20 transcriptome annotation using kallisto [64], and normalized expression data in transcripts per million (TPM) was obtained (Table S1).

**Fig. 2:**
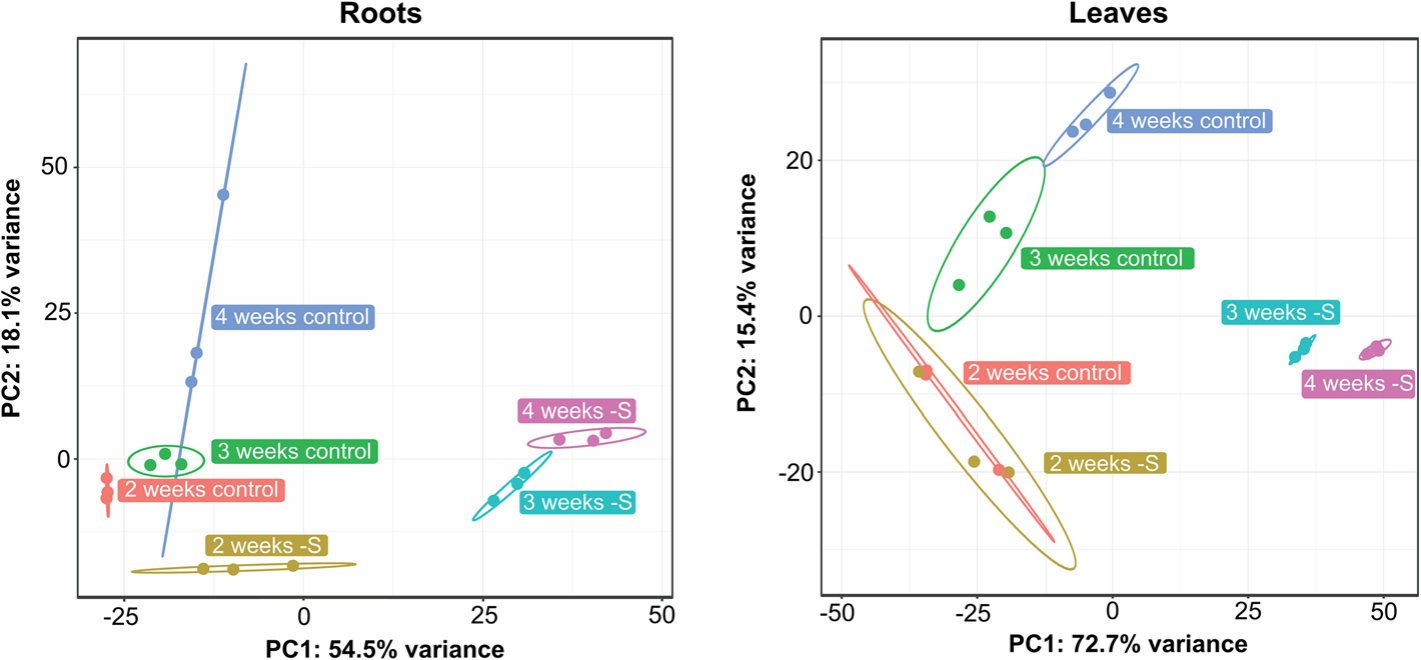
Exploratory data analysis of tomato RNA-seq shows that the response to sulfate starvation begins in root tissue. Principal Component Analysis (PCA) of RNA-seq data of roots (left) and leaves (right) samples. PCA analysis was performed with pcaExplorer R package [65] using log_2_-transformed normalized expression data. Ellipses represents the 95% confidence interval of 3 independent experiments. Replicates of the same experiment are indicated with the same color

Principal component analysis (PCA) of the RNA-seq data showed that samples are well separated along the first component, which explains more than 50% of the variability in leaf and root samples (Fig. 2). Interestingly, full nutrient and sulfate-starved samples from leaves are grouped together at 2 weeks after sowing, indicating similar transcript expression profiles at this time point (Fig. 2). In contrast, control and sulfate-starved samples from root tissue at 2 weeks are well separated, suggesting that sulfate starvation alters the plant transcriptome in an organ- and time-dependent fashion, with an earlier response in root tissue.

Accordingly, no genes were found differentially expressed between control and sulfate-starved leaves at 2 weeks after sowing, while 438 genes were differentially expressed in roots in this time point (log_2_ Fold Change> 1 and q-value< 0.05) (Figure S2A and Table S2). In addition, 55% of these 438 genes are subsequently regulated in leaves (241 genes, Figure S2B), which point out an orchestrated whole organism response to sulfate deprivation over developmental time.

A considerable number of genes were differentially expressed by sulfate starvation in roots and leaves: 7024 genes at 3 weeks after sowing and 6163 at 4 weeks after sowing, with most genes being differentially expressed in leaves (Figure S2A and Table S2). This major reprogramming of the plant transcriptome correlates with the observed differences in leaf and root growth of control and sulfate-deprived plants between weeks 3 and 4, suggesting changes in gene expression are partly responsible for the observed phenotypes (Fig. 1a and b).

**Fig. 3:**
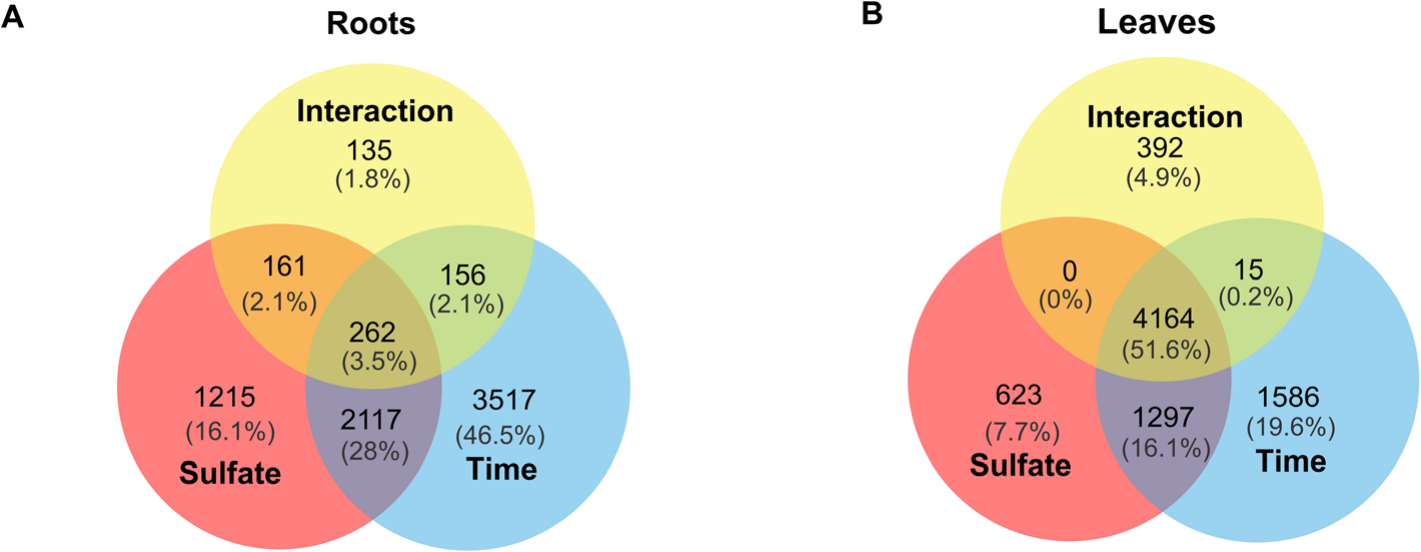
The impact of sulfate starvation on the transcriptome depends on the age of the plants in leaves but not in roots. Venn diagram showing genes significantly regulated by sulfate, time or by interaction of both factors in roots (**a**) and leaves (**b**). Multifactorial analysis was performed using sleuth R package [66] with a q-value < 0.05

In order to identify genes that are differentially expressed in response to sulfate starvation, time or a sulfate starvation-time interaction, we performed a multifactorial analysis of RNA-seq data in each tissue using sleuth [66]. In the case of roots, 6052 genes were significantly affected by time, 3755 genes by sulfate starvation and 714 by the interaction of both factors (q-value < 0.05, and log_2_FC > 1) (Fig. 3a). In the case of leaves, 6084 genes were affected by sulfate starvation, 7062 genes by time and 4571 genes by the interaction of both factors (q-value < 0.05, and log_2_FC > 1) (Fig. 3b). In contrast to root tissue, these results indicate that the response to sulfate starvation in leaves has a strong dependence on the plant age. In fact, about 90% of sulfate-responsive genes in leaves are significantly modulated by time (Fig. 3b).

To validate the results of the RNA-seq analysis with an alternative RNA quantification methodology, we selected 10 sulfate-regulated genes in root and leaves involved in different molecular and biological functions including sulfate metabolism, transport and transcriptional regulation, as well as a group of genes with unknown functions (Table S3). The expression patterns obtained by qPCR analysis showed a significant positive correlation with RNA-seq data (*P* < 0.0001; 0.89 Pearson correlation value; Figure S3), indicating that most of genes analyzed showed a similar expression pattern than the ones identified by RNA-seq analysis.

### Comparative analysis of the tomato and Arabidopsis sulfate starvation-responsive transcriptome

**Fig. 4:**
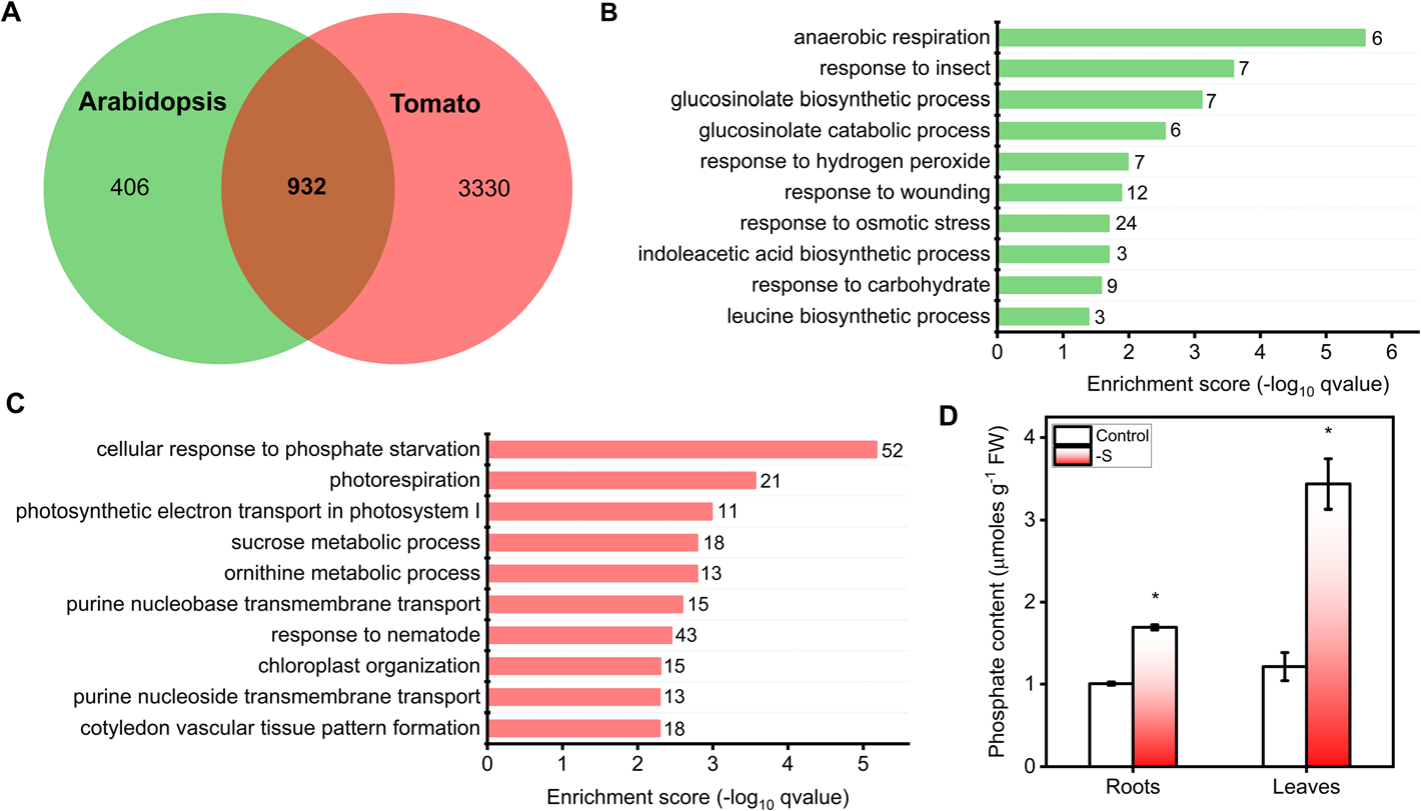
Comparative transcriptome analysis of Arabidopsis and tomato plants in response to sulfate starvation reveal conserved and specific features. **a** Venn diagram showing sulfate-responsive genes shared between Arabidopsis and tomato plants. The plant comparative genomics resource PLAZA 4.0 was used to identify the orthologous genes. The Arabidopsis sulfate-responsive genes were obtained from a meta-analysis [43]. **b** GO term enrichment analysis of genes exclusively regulated by sulfate in Arabidopsis. The 10 most over-represented biological functions are shown along with the number of genes belongs to each GO term. **c** GO term enrichment analysis of genes exclusively regulated by sulfate in tomato plants. The 10 most over-represented biological functions are shown along with the number of genes belongs to each GO term. **d** Sulfate starvation increase phosphate content in tomato roots and leaves of plants grown under full nutrient or S-limiting conditions for 3 weeks. Phosphate content was determined by the malachite green assay [69]. A Student’s t-test was performed to test the significance (*P* < 0.05) of the differences between sulfate-starved and control samples (*). Values plotted correspond to the means of three independent experiments ± standard deviation. 3–5 different plants were measured for each experimental replicate

In a previous study, we identified 2046 genes regulated by sulfate availability in *Arabidopsis thaliana* using an integrative meta-analysis of transcriptomic data [43]. This meta-analysis included samples of roots and leaves at different stages of development (from seedling to juvenile plants). In the case of tomato plants, multifactorial analysis identified 7589 sulfate-responsive genes in roots and leaves. In order to compare the sulfate starvation responsive transcriptome of both Arabidopsis and tomato, we first performed an orthology analysis of sulfate responsive genes. The identification of orthologous groups allows cross-referencing of genes from multiple species [67]. We used the PLAZA 4.0 database [68] to identify the ortholog group to which each sulfate-regulated gene belongs. Thus, we identified 4262 orthologous gene families out the 7589 sulfate-responsive genes in tomato and 1338 orthologous gene families out the 2046 sulfate-responsive genes in Arabidopsis (Table S4). We found that 70% of the Arabidopsis orthologous gene families that are associated with sulfate-responsive genes were also present in tomato plants (Fig. 4a). This result indicates that the RNA-seq analysis in tomato plants captured most of the sulfate-responsive gene families previously detected in Arabidopsis.

Over-representation analysis of biological functions shared by both species revealed several GO terms that are expected to be conserved in the sulfate response as “cellular oxidant detoxification”, “sulfate transmembrane transport” or “sulfur utilization” (Table S5). Interestingly, we also found a significant enrichment (adjusted *p*-value < 0.05) in GO terms that have not been functionally analyzed in previous studies in the context of the sulfate starvation response such as those related to cell wall or cytokinin transport (Table S5). On the other hand, specific GO terms of the Brassicaceae family such as GSL biosynthesis are among the most over-represented biological processes in the case of sulfate-responsive genes in Arabidopsis (Fig. 4b and Table S5). The intersection between the lists of orthologue genes of both species also detected sulfate-responsive processes that are found exclusively in tomato plants. The most over-represented biological process in this group of genes was “cellular response to phosphate starvation” (Fig. 4c and Table S5), suggesting that sulfate deficiency also affects internal phosphate levels in tomato plants. Specifically, we found 52 genes in this biological process including *SPX* genes [70], *phosphatases* and genes encoding enzymes involved in phosphate mobilization by membrane lipid remodeling such as UDP-sulfoquinovose synthase [71, 72] (Figure S4). In order to determine the response of this set of phosphate-related genes to sulfate starvation in tomato plants, we computed the average fold of change between sulfate starved and control samples in both tissues and we then performed a hierarchical clustering analysis. We found that 60% of the genes involved in the phosphate starvation response (30 out 52 genes) showed lower expression levels in sulfate-starved plants at 3 and 4 weeks after sowing in both tissues (Cluster 3, Figure S4), suggesting that sulfate starvation alters internal phosphate levels. To address this hypothesis, we determined the internal phosphate levels at 3 weeks after sowing in both tissues. The results shown in Fig. 4d, indicate that sulfate-starved plants exhibit significantly higher phosphate levels than control plants in both tissues, with a higher phosphate accumulation in leaves.

### Temporal dynamics of gene expression of tomato roots and leaves are altered by sulfate starvation

**Fig. 5:**
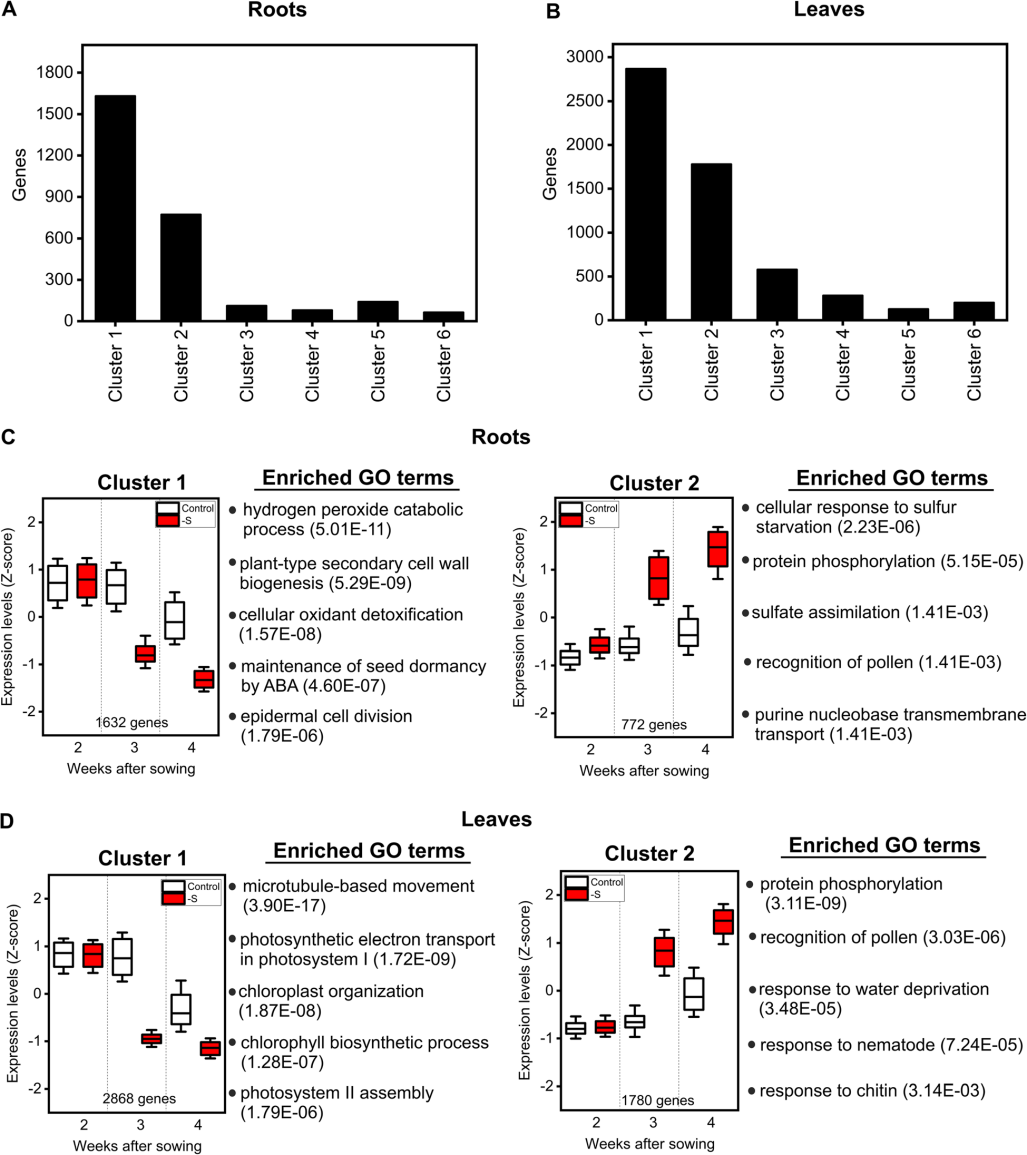
Gene co-expression clusters of roots and leaves during tomato development under sulfate starvation. Co-expression clusters of genes exclusively regulated by sulfate and time in roots (**a**) or leaves (**b**). Expression patters of major clusters in roots (**c**) and leaves **(d**). On each box, the central mark indicates the median, and the bottom and top edges of the box indicate the 25th and 75th percentiles, respectively. Whisker indicates standard deviation of the expression data of all genes belongs to the cluster. The top 5 enriched GO terms are indicated to the right of each box plot with the q-values in brackets

In order to get further insights about the temporal patterns of expression of the genes and associated biological processes affected by sulfate starvation, we computed Pearson correlation indexes for each pair of sulfate and time-regulated genes in roots and leaves. We performed a hierarchical clustering analysis using Dynamic TreeCut [73], and identified six co-expression clusters for genes regulated by sulfate and time in roots and leaves (Fig. 5a, Fig. 5b, Figure S5, Figure S6). We found that the majority of genes (60% in roots and 72% in leaves) are contained in Clusters 1 and 2 (Fig. 5a and Fig. 5b). We thus decided to analyze these clusters in more detail.

As shown in Fig. 5c and Fig. 5d, the expression profiles of Clusters 1 and 2 in roots and leaves show similar trends. Genes in Cluster 1 are repressed over time, with an earlier decrease in mRNA levels under sulfate starvation conditions, between weeks 2 and 3. On the other hand, the expression of genes in Cluster 2 slightly increases between weeks 3 and 4 in control conditions, while an important increase in gene expression is shown between weeks 2 and 3 under sulfate starvation conditions (Fig. 5c and Fig. 5d). Although the expression patterns of the genes contained in Clusters 1 and 2 are similar between roots and leaves, the identity of these sulfate-responsive genes differs between these organs (Table S6 and Table S7). As such, we found different enriched biological processes associated with Clusters 1 and 2 in roots and leaves. In roots, Cluster 1 is enriched in genes associated to growth, such as “epidermal cell division” and “plant-type secondary cell wall biogenesis”, as well as processes related to redox activity, such as “hydrogen peroxide catabolic process” and “cellular oxidant detoxification” (Fig. 5c and Table S8). In leaves, we found an enrichment of genes involved in photosynthesis, microtubule-based movement and several GO terms related with cell division such as “mitotic cell cycle checkpoint” or “mitotic spindle organization” (Table S9). GO terms related to photosynthesis are especially abundant in this cluster, so we analyzed in more detail the distribution of these genes across different categories of primary metabolism using Mapman4 annotation framework [74]. In the case of photosynthesis, most genes are involved in light reactions, followed by the Calvin-Benson cycle and sucrose/starch metabolism (Figure S7A). Cluster 1 genes are also involved in other aspects of primary metabolism such as nitrogen assimilation and amino acid biosynthesis as well as fatty acid biosynthesis (Figure S7A). A similar result was obtained when all differentially expressed genes by sulfate starvation in leaves were analyzed using Mapman4 (Figure S7B). On the other hand, genes related to photosynthesis were under-represented in roots (Figure S7C), while other metabolic processes such as cell wall, lipid or amino acid metabolism showed a similar representation in roots and leaves (Figure S7B and Figure S7C). In the case of Cluster 2, and consistent with the marked induction in gene expression triggered by sulfate starvation shown in this cluster, we found an overrepresentation of genes involved in biological processes related to the cellular response to S starvation such as *APR* genes (Solyc02g032860, Solyc02g080640 and Solyc03g031620), *RESPONSE TO LOW SULFUR* (*LSU*) genes (Solyc03g096760, Solyc03g096770, Solyc03g096780) or *sulfate transporters* (Solyc05g054740, Solyc04g054730, Solyc04g072760, Solyc12g056930) (Fig. 5c and Table S8). Cluster 2 in leaves is enriched in GO terms related with the response to stress and protein phosphorylation (Fig. 5d and Table S9). Interestingly, we did not find an overrepresentation of S-starvation-related processes in Cluster 2 in leaves, as observed in roots. In fact, classical genes involved in the sulfate starvation response such as *SULTR*, *ATPS, APR* or *LSU* belong to Cluster 6 (Figure S6), suggesting an organ-specific expression of these genes in response to external S during development.

In summary, sulfate starvation promotes significant changes in the temporal expression patterns of a large number of genes in roots and leaves, of which many are associated with functions related to cellular growth, photosynthesis and sulfate uptake and metabolism. Moreover, the comparative analysis of major gene co-expression clusters between roots and leaves indicates an organ-specific response of the tomato plants to sulfate starvation.

### Identification of transcription factors involved in the sulfate starvation response in tomato

Our results point at major transcriptomic changes occurring in tomato roots and leaves in response to sulfate starvation. As described in Arabidopsis, some of these changes may be attributed to transcriptional regulation by TFs. Although some TFs controlling sulfate responses have been identified in Arabidopsis [31], to date there is no information about sulfate-responsive TFs and their role in responses to sulfate starvation in tomato. In order to identify key TFs controlling the response to sulfate starvation of tomato, we constructed gene regulatory networks for roots and leaves using co-expression data and regulatory information on TF-DNA interaction obtained from PlantRegMap [75] .

In the case of roots, we obtained a gene regulatory network consisting of 24 TFs and 172 targets (Figure S8 and Table S10). TFs of the root regulatory network belong to 17 different families (Table S11), with MYB and bZIP TFs being the most abundant families (4 and 3 members, respectively). In the same way, we constructed a TF-target network using the identified sulfate-regulated genes in leaves which was composed by 45 TFs and 566 targets (Figure S9 and Table S12). Specifically, ERF, TCP and WRKY were the most abundant TF families in the leaves network with 7 and 5 members, respectively (Table S11). Notably, the most abundant TF families in the regulatory network of leaves were not shared with the root network (Table S11), suggesting organ-specific regulation of the transcriptional response to sulfate starvation.

**Fig. 6:**
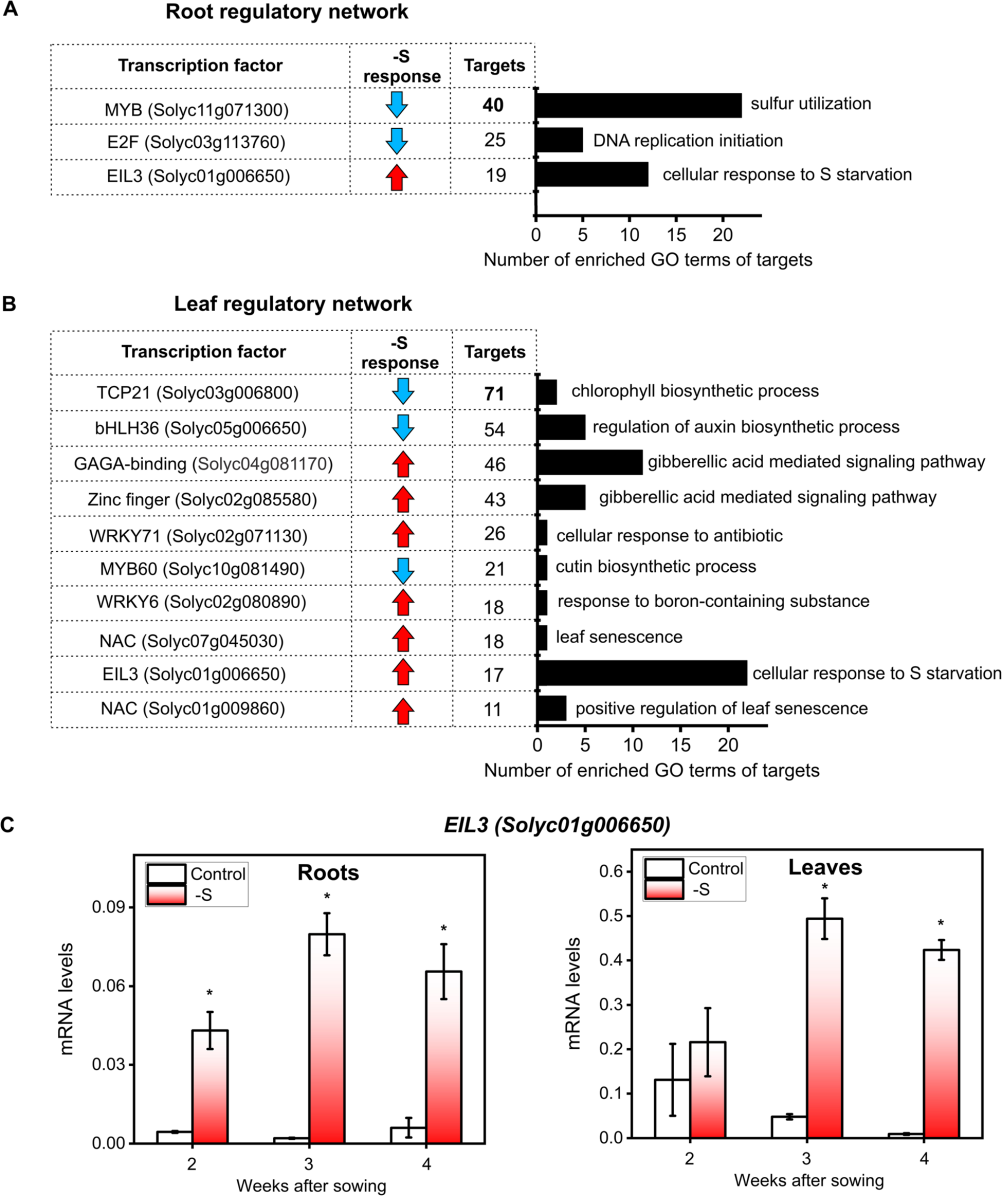
A gene regulatory network analysis identifies new TFs involved in the sulfate starvation response in tomato plants. **a**. TFs from the root regulatory network that have over-represented biological functions in their target genes. The root regulatory network was constructed considering TF-target interaction according to the information available in PlantRegMap and Pearson correlation threshold > 0.9 in root samples. **b** TFs of the leaf regulatory network with over-represented biological functions in their target genes. TFs and their potential targets were identified using the information available in PlantRegMap and Pearson correlation threshold > 0.9 in leaf samples. **c** RT-qPCR analysis of *EIL3* TF (Solyc01g006650) in tomato plants. A Student’s t-test was performed to test the significance (*P* < 0.05) of the differences between sulfate-starved and control samples (*). Values plotted correspond to the means of three independent experiments ± standard deviation. 3–5 different plants were measured for each experimental replicate

We next focused our analysis on TFs whose predicted target genes have overrepresented biological functions (adjusted *p*-value < 0.05), in order to correlate TF regulation to changes in functional processes. As shown in Fig. 6a and Fig. 6b, we found 3 TFs in roots and 10 TFs in leaves that potentially control genes involved in the sulfate starvation response, phytohormone biosynthesis and signaling, senescence, among other biological processes. Most connected factors in terms of outdegree were a MYB TF (Solyc11g071300) in roots and TCP 21 TF (Solyc03g006800) in leaves, showing 40 and 71 targets respectively (Fig. 6a and Fig. 6b). Notably, MYB target genes are involved in S utilization, while TCP21 ones are involved in photosynthesis (Fig. 6a and Fig. 6b, Table S13).

As mentioned above, our results discovered important changes in gene expression in response to sulfate starvation, suggesting the involvement of different sets of TFs acting in both roots and leaves. However, we found one TF shared by both root and leaf gene regulatory networks which is early induced in roots but has a later response in leaves (Fig. 6c). This TF is member of the EIL family and corresponds to ETHYLENE INSENSITIVE 3-LIKE 3 (EIL3, Solyc01g006650). The closest Arabidopsis gene in terms of amino acid identity is SLIM1, which is a key regulatory factor of the sulfate starvation response [23]. In addition, GO enrichment analysis showed that the identified tomato EIL3 TF might control the expression of a set of genes related to sulfate starvation, including classical sulfate-responsive genes such as *LSU*s as well as genes encoding enzymes involved in the reductive steps of sulfate assimilation (Table S14). Furthermore, we found that 60% of the tomato TF target genes are shared with targets predicted for Arabidopsis *SLIM1,* according to PlantRegMap, including sulfate-response genes such as *LSU*s and *SDI*s (Table S14). Overall, we identified several candidate TFs to mediate the transcriptome reprogramming that occurs during sulfate starvation response in roots and leaves, highlighting the possible key regulatory role of the EIL3 TF in tomato.

## Discussion

### Temporal and organ-specific transcriptomic response to sulfate starvation in tomato plants

Our current understanding of the transcriptomic response of plants to sulfate availability is mostly based on knowledge gathered in the model plant *Arabidopsis thaliana* [31]. Most of the analyses carried out in Arabidopsis do not contain whole transcriptome information since they were performed using microarray technology [31, 43]. On the other hand, the tissue-specific response to sulfate availability over time has been poorly explored so far and most studies have been performed at the seedling stage in Arabidopsis. Moreover, while transcriptomics approaches have been utilized to uncover the sulfate deficiency response in crops such as wheat [39–42], no transcriptome-wide analysis of the sulfate deficiency response is available for tomato. In this work, we report a comprehensive transcriptome analysis of the sulfate starvation response of tomato plants using RNA-seq, with a temporal and organ resolution.

We found that sulfate starvation has a strong impact on the growth of roots and leaves, starting from 3 weeks after sowing. The inhibition of growth was accompanied with drastic changes in gene expression in both organs. Analysis of enriched GO terms associated with sulfate-responsive genes allowed us to discover several biological processes related to plant growth that were altered by sulfate starvation. To date, several studies have demonstrated that plants activate sulfate uptake by inducing the expression of high-affinity sulfate transporters in response to sulfate starvation [15]. Moreover, it is well established that plants activate the sulfate assimilatory pathway and the degradation of glutathione and other S compounds such as GSLs during sulfate starvation [15]. As expected, genes involved in sulfate transport and metabolism are differentially expressed according to external sulfate availability in tomato plants, being induced over time in response to sulfate starvation. Specifically, we identified 12 sulfate transporter genes, 3 *ATPS* and 3 *APR* genes significantly regulated by sulfate starvation in at least one sample (q-value < 0.05 and log2FC > 1) (Table S15).

Our expression analysis also identified several genes involved in cell wall biosynthesis and modification, whose expression was altered by sulfate deprivation. It is well known that cell wall is a crucial component for plant biomass accumulation and cell expansion, which are complementary processes that sustain plant growth [76]. However, how nutrient availability regulates cell wall biosynthesis and organization remains poorly understood [77]. In this work we found that the expression of 6 *XET* genes decreases over time in sulfate-starved plants (Cluster 1, Table S6). *XET* genes code for enzymes that have the capacity to loosen cell walls, affecting cell expansion and growth [78]. Thus, repression of *XET* genes by sulfate starvation can partly explain the observed phenotypes. Accordingly, XET knock-out mutants have reduced cell sizes [79–82], and overexpression or exogenous application of XET proteins decrease cell expansion [83], suggesting that the regulation of *XET* genes by sulfate starvation might partly explain the observed phenotypes.

Our expression analyses also reveal a significant level of organ-specific response related to the growth inhibition phenotype. Sulfate starvation triggers a strong decrease in the transcript levels of genes related to photosynthesis in leaves, including those related to light and carbon reactions. Taking into account the well-known relationship between plant growth and photosynthesis [84], lower expression of photosynthetic genes likely translates into decreased growth as we observed in the case of sulfate starvation. Interestingly, a similar inhibitory effect of the sulfate starvation on photosynthesis and growth has been demonstrated in other photosynthetic organisms such as microalgae *Dunaliella salina* [85] and *Chlamydomonas reinhardtii* [86], suggesting that the regulation of photosynthetic genes by sulfate starvation might be conserved in photosynthetic organisms.

### Phosphate levels are regulated by sulfate starvation in tomato

In order to identify novel and specific biological processes in the response to sulfate starvation of tomato plants, we intersected the orthologous groups of genes regulated by sulfate availability in this work with those reported in a previous meta-analysis in Arabidopsis [43]. Remarkably 70% of the Arabidopsis sulfate-responsive orthologous genes were also regulated by this nutrient in tomato (Fig. 4a), indicating a conserved response to sulfate between Arabidopsis and tomato plants. On the other hand, we also found a group of orthologous genes exclusively regulated by sulfate in tomato plants, which showed over-represented biological process including “cellular response to phosphate starvation” “photorespiration”, “photosynthetic electron transport in photosystem”, “ornithine metabolic process”, “sucrose metabolic process”, among others (Fig. 4c and Table S5), suggesting additional mechanisms might be involved in sulfate starvation responses in tomato.

Remarkably, the most over-represented biological process of orthologous genes regulated by sulfate in tomato plants was “cellular response to phosphate starvation”, suggesting that sulfate deficiency affects the internal phosphate levels in tomato plants. In agreement with this result, phosphate content analysis revealed an increase of this nutrient in sulfate-starved plants in both organs. A similar alteration of phosphate levels by sulfate starvation has been reported in Arabidopsis [10, 11], with plants showing a 3-fold increase of free phosphate in response to S-deficiency. This increase in phosphate levels is not triggered by deficiency of other nutrients such as potassium or iron [10], indicating a specific interaction of sulfate and phosphate homeostasis.

The increase of internal phosphate levels can be achieved by an activation of phosphate transport during sulfate starvation, as has been previously demonstrated in Arabidopsis [11]. Although we found a higher phosphate content in both organs in sulfate-starved plants, phosphate accumulation was higher in leaves, suggesting an increased translocation of phosphate from roots to leaves. In Arabidopsis, phosphate transport from root to shoot is mediated by the PHO1 transporter [87], whose expression is controlled by phosphate starvation at the transcriptional and post-translational levels [9]. PHO1 together with PHT1;9, a member of the PHT1 family of phosphate transporters, are involved in the increased phosphate translocation from roots to shoots during sulfate starvation in Arabidopsis [11]. We found that several members of the PHO1 and PHT1 gene families (Solyc02g088220, Solyc09g066410 and Solyc09g090080; Figure S10) are induced by sulfate starvation in tomato, suggesting that sulfate starvation promotes phosphate transport from roots to leaves in tomato. These results are consistent with previous works in potato and tomato showing that transcript levels of the phosphate transporters StPT2 and LePT1 increase under sulfate starvation [88, 89].

In Arabidopsis, the transcript levels of the main *phosphate uptake transporters (PHT1;1, PHT1;2, and PHT1;4*) and *PHO1* genes are not affected by sulfate starvation [11], which suggest significant differences in the mechanism of control of phosphate allocation between Arabidopsis and tomato during sulfate starvation. The biological role of increasing phosphate levels in response to sulfate starvation is not fully understood. A rapid replacement of sulfolipids by phospholipids has been reported to occur during the early stage of sulfate starvation [90]. This increased demand of phosphate might require an enhanced acquisition of this nutrient in the later stages of sulfate starvation response. Recent work in Arabidopsis has shown that the re-addition of sulfate to the medium restores phosphate levels in shoots and xylem sap [11], indicating a direct relation between sulfate and phosphate levels. However, further research is required to unravel the complex crosstalk between sulfate and phosphate starvation.

### Identification of key TFs involved in response to sulfate-starvation in tomato plants

Gene regulatory network analysis integrating gene expression data and regulatory TF-target data allowed us to identify candidate TFs involved in the response to sulfate starvation during early development of tomato plants. Among the TFs regulated by sulfate starvation in both tissues, we identified a member of the EIL family, whose closest Arabidopsis homolog is SLIM1, a key regulatory factor in the sulfate starvation response [23]. Transcriptome analysis of *slim1* mutants showed lower extent of up-regulation of sulfate transporters and lower extent of down-regulation of GSL genes compared to WT plants [23]. Moreover, SLIM1 is required for plant growth under sulfate starvation conditions [23]. Notably, SLIM1 homologs from rice have been reported to complement the *slim1* mutants from Arabidopsis, indicating a functional conservation of this group of EIL TFs in controlling the sulfate starvation response [23]. Our network analysis highlighted that *EIL3* might control an important group of genes involved in sulfate transport and metabolism, like Arabidopsis SLIM1 (Table S14). Specifically, we found that 60% of the predicted targets of the tomato *EIL3* are also described as SLIM1 ones.

Interestingly, *SLIM1* transcript or its subcellular localization are not controlled by sulfate deficiency [23], however a recent report shows that SLIM1 can interact with E3 ubiquitin ligases, suggesting SLIM1 ubiquitination and protein degradation might have a role in controlling SLIM1 function in S metabolism [91]. In contrast, we found that the transcript levels of tomato *EIL3* are dramatically increased by sulfate starvation in roots and leaves of tomato plants. However, we cannot rule out that sulfate starvation might also control EIL3 function at the post-translational level. Further work is therefore required to assess the role of EIL3 in the sulfate response of tomato plants.

Besides EIL3, our analysis identified a Myb TF (Solyc11g071300) as the most connected TF of the root regulatory network. According to the functional analysis of its target genes, the identified Myb TF might be involved in the control of cell division, which is an important biological process for plant growth. In agreement, Arabidopsis orthologues MYB3R-1 and MYB3R-4 act as transcriptional activators regulating cytokinesis [92]. In fact, 53.3% of the genes from the G2/M-specific class were downregulated in double mutant Arabidopsis plants. Besides, it was uncovered that both MYB3R4 TFs bind to MSA motifs in vitro, which are common cis-elements of G2/M phase-specific genes [93]. All these data suggest that the MYB TFs might play an important role controlling cell division under sulfate starvation conditions.

On the other hand, the most connected TF of the leaf regulatory network was TCP21 (Solyc03g006800). According to the GO enrichment analysis of its target genes, this TF is related to photosynthesis, which is an essential metabolic process for vegetative growth. Interestingly, several TCP TFs in Arabidopsis have been shown to regulate leaf growth and development [94]. The closest homolog gene of the tomato TCP in Arabidopsis is TCP19, which is involved in the control of leaf senescence in a redundant manner with TCP20 [95]. Our transcriptomic analysis shows that the expression of the identified tomato TCP is reduced during sulfate starvation in leaves along with genes related to photosynthesis, leading to a strong reduction in plant growth. Therefore, TCPs might play important functions in the control of leaf growth during sulfate starvation in tomato plants.

## Conclusions

In summary, our results shed lights into the extensive transcriptome reprogramming that is elicited during sulfate starvation in tomato roots and leaves and highlight the role of TFs such as MYB, TCP and EIL3 in coordinating this complex response. We hope this work can serve as a basis for further studies addressing the role of specific genes and processes to further our understanding of the regulatory mechanisms underlying sulfate starvation responses in tomato and other crops.

## Methods

### Plant material and growth conditions

Seeds of the *Solanum lycopersicum* ‘Moneymaker’ cultivar (obtained commercially from Semilias, Chile) were grown for 4 weeks in 0.5X Murashige and Skoog (MS) salts [96] (pH 5.7) using rockwool as support material. This liquid culture medium was replaced twice a week to maintain constant nutrient concentrations. For plants grown in sulfate starvation conditions, sulfate salts contained in the MS medium were replaced with equivalent chloride salts [97]. Tomato plants were cultivated in a controlled growth cabinet (Bioref-19, Pitec, Chile) with LED lights (200 μmol m^− 2^ s^− 1^) at 22 °C under 16-h-light/8-h-dark cycles. Three independent experiments were performed using 3 to 5 different plants per replicate.

### Plant growth quantification

For determination of growth-related parameters, leaves and roots were scanned using an Epson Perfection V600 photo scanner. Total leaf area and primary root length were determined from scanned images using the ImageJ software (v1.52) (https://imagej.nih.gov/ij/). Total root and leaf fresh weights were determined using an analytical balance.

### Library construction and RNA-Seq data analysis

Leaves and roots samples were frozen in liquid nitrogen at 2, 3 and 4 weeks after sowing and RNA was isolated using the mirVana miRNA Isolation Kit (Invitrogen), following the manufacturer’s instructions for total RNA isolation. The extraction was subjected to on-column DNase I treatment (TURBO DNase, Invitrogen). One microgram of DNase I-treated RNA was used to generate poly-A-enriched sequencing libraries using the TruSeq Stranded mRNA Library Prep kit (Illumina). Libraries were sequenced as 75-nt single-end reads on a NextSeq500 system (Illumina).

The sequenced reads were quality-checked using FastQC version 0.11.5. Taking into account the high quality of the sequenced reads, we skipped the trimming process in order to avoid any potential biases as has been previously described [98]. To quantify the mRNA levels of annotated genes, sequenced reads were pseudo-aligned to the publicly available *Solanum lycopersicum* transcriptome using kallisto (v0.44) [64]. Transcript indices for kallisto were generated from tomato annotation version ITAG3.2 (https://solgenomics.net/organism/Solanum_lycopersicum/genome) which included 35,768 cDNAs. On average, each sample had 30 million reads, of which 27 million reads (90%) were pseudo-aligned to the tomato transcriptome. The estimated read counts and calculated transcripts per million (TPM) were used for differential expression analysis [66]. Differential expression analysis of the sulfate starvation response in each time and organ was performed using the likelihood ratio test (LRT) implemented in sleuth (v.0.30.0) [66] with three independent replicates. We defined differential gene expression by setting an arbitrary q-value threshold of 0.05 and absolute log_2_ Fold Change (FC) > 1.

We performed a multivariate linear model to test whether the expression of a given gene could be explained by the sulfate availability, time or the interaction of both factors using the R package sleuth (v.0.30.0) [66]. In order to identify significantly regulated genes for each factor, we also applied a q-value threshold of 0.05 and absolute log_2_ FC > 1 in at least one level of each factor. The identification of gene co-expression clusters associated to each factor was performed using the R package dynamicTreeCut (v.1.63–1) [73], which is a hierarchical clustering method for automatic identification of gene co-expression clusters. Principal component analysis (PCA) was performed on the expression data using pcaExplorer (v.2.14.0) [65].

### Gene ontology enrichment analysis

Gene Ontology (GO) enrichment analyses were performed using BiNGO (v.3.0.3) [99] with *Solanum lycopersicum* GO annotations obtained from PLAZA 4.0 [68]. Benjamini and Hochberg false discovery rate (FDR) correction (< 0.05) was applied on the over-represented GO terms obtained in the respective gene set after performing a hypergeometric test with BiNGO. We filtered out the GO terms with less than three annotated genes and used only GO terms associated to biological processes. To reduce the redundancy between GO terms, we selected the overrepresented GO terms with the higher level in the hierarchical GO tree obtained with BiNGO.

### Comparative transcriptomics analyses

In order to determine the sulfate-starvation responsive genes that are shared between Arabidopsis and tomato, we used the ortholog database PLAZA 4.0 [68] to assign a common identification code for the genes of both species (orthologous gene family ID). We intersected both lists using VennPainter software [100] to identify conserved and species-specific sulfate-responsive gene families. The group of Arabidopsis sulfate-responsive genes was obtained from a recent transcriptomic meta-analysis by our group [43]. In the case of tomato, the set of sulfate-responsive genes was obtained from the RNA-seq analysis performed in this work, considering genes significantly regulated by sulfate factor or by the interaction of sulfate and time in roots or leaves (q-value threshold of 0.05 and absolute log_2_ FC > 1).

### Gene regulatory network analysis

We constructed a gene regulatory network for each organ considering the following criteria: first, we only included in this analysis genes that showed a significant regulation by sulfate or by the interaction of sulfate and time factors (q-value < 0.05 and absolute log_2_ FC > 1). Second, we only considered genes that have evidence of TF-target interaction according to the PlantRegMap [75] in order to predict direct regulations. Third, pairs of genes with a TF-target interaction must have a Pearson correlation greater than 0.9 to obtain similar expression profiles between TFs and their target genes. In this manner, we focused on coherent temporal responses of TFs and their targets to sulfate starvation. Pearson correlation was determined from the RNA-seq data obtained in each tissue using the R package rsgcc (v.1.0.6) [101].

Gene regulatory networks were visualized and analyzed using Cytoscape 3.7 [102]. Candidate TFs were selected considering their connectivity and overrepresented GO terms of their target genes.

### Quantitative real-time PCR (qPCR)

For qPCR analyses, cDNA was generated from 500 ng of total RNA using 5X All-In-One RT MasterMix (Applied Biological Materials, Canada) following the manufacturer’s instructions. qPCR reactions were performed with 25 ng of cDNA using the PowerUp SYBR Green Master Mix (Applied Biosystems™) in a CFX96 Real-Time PCR Detection System (Bio-Rad). Raw fluorescent data was analyzed using the Real-time PCR Miner 4.0 software [103] to obtain cycle threshold values and gene amplification efficiencies. The expression of each target gene of interest was normalized by the Actin-7 gene (Solyc11g005330), which showed stable mRNA levels across all samples analyzed by RNA-seq. Sequences of the qPCR primers used in this study are provided in Table S16.

### Sulfate and phosphate content analysis

Sulfate content was quantified using a turbidimetric method described by Tabatabai and Bremner [69, 104]. Phosphate content was determined using the Malaquite Green Phosphate Assay Kit (Sigma-Aldrich, Catalog Number MAK307) according to the manufacturer’s recommendations.

## Supplementary information

**Additional file 1: Figure S1.** Total leaf area is reduced by sulfate deficiency from 3 weeks after sowing. **Figure S2.** A) Number of differentially expressed genes by sulfate starvation in roots, leaves or shared by both organs at 2, 3 and 4 weeks after sowing. B) Venn diagrams showing the overlap between early sulfate-regulated genes in roots and late regulated genes in leaves. **Figure S3.** qPCR validation of RNA-seq data. A) Correlation between relative mRNA levels of qPCR and RNA-seq data on selected transcripts. The list of selected differentially expressed genes by sulfate starvation is shown in Table S3. B) Heatmap comparing the expression patterns of selected genes analyzed by qPCR and RNA-seq. **Figure S4.** Hierarchical clustering of 52 genes included in the GO term “cellular response to phosphate starvation”, the most enriched GO term of sulfate-responsive orthologous gene families that are found exclusively in tomato plants. **Figure S5.** Co-expression clusters down-regulatedby sulfate starvation of genes significantly affected by sulfate and time in roots (left) and leaves (right) (q-value< 0.05 and absolute log2 FC > 1). On each box, the central mark indicates the median, and the bottom and top edges of the box indicate the 25th and 75th percentiles, respectively. Whisker indicates standard deviation of the expression data of all genes belongs to the cluster. **Figure S6.** Co-expression clusters up-regulated by sulfate starvation of genes significantly affected by sulfate and time in leaves (q-value< 0.05 and absolute log_2_ FC > 1). On each box, the central mark indicates the median, and the bottom and top edges of the box indicate the 25th and 75th percentiles, respectively. Whisker indicates standard deviation of the expression data of all genes belongs to the cluster. **Figure S7.** A) Distribution of Cluster 1 genes regulated by sulfate and time in leaves across different categories of the primary metabolism using Mapman4 annotation framework. B) Mapman analysis of sulfate starvation responsive genes in at least one time in leaves. C) Mapman analysis of sulfate starvation responsive genes in at least one time in roots. **Figure S8.** The root regulatory network. Blue nodes represent TFs and red nodes represent target genes. **Figure S9.** The leaf regulatory network. Blue nodes represent TFs and red nodes represent target genes. **Figure S10.** Expression profiles of the phosphate transporter genes *Solyc02g088220*, *Solyc09g066410* and *Solyc09g090080* in response to sulfate starvation at 2, 3 and 4 weeks after sowing.

**Additional file 2: Table S1.** Normalized RNA-seq data in transcripts per million (TPM) scale. **Table S2.** Differentially expressed genes between sulfate-starved and control tomato plants at 2, 3 and 4 weeks after sowing. Differential expression analysis was performed in roots and leaves using the sleuth R package (q-value< 0.05 and absolute log2 FC > 1). **Table S3.** List of genes validated by qPCR indicating whether the sulfate regulation was previously reported in Arabidopsis. **Table S4.** List of Arabidopsis and tomato sulfate-responsive orthologous genes. **Table S5.** Enriched GO terms for orthologous sulfate-responsive genes in Arabidopsis, tomato, or shared in both species. **Table S6**. Genes exclusively regulated by sulfate and time in roots. Multifactorial analysis was performed using the sleuth R package (q-value< 0.05 and log2 FC > 1 in at least one level of each factor). **Table S7.** Genes exclusively regulated by sulfate and time in leaves. Multifactorial analysis was performed using the sleuth R package (q-value< 0.05 and log2 FC > 1 in at least one level of each factor). **Table S8.** Enriched GO terms for genes of root clusters. Only significant GO terms (adjusted *p*-value < 0.05) within the category of biological process are included in this table. **Table S9.** Enriched GO terms for genes of leaf clusters. Only significant GO terms (adjusted p-value < 0.05) within the category of biological process are included in this table. **Table S10.** List of TF-target interactions and nodes annotation of the root regulatory network. **Table S11.** Summary of the transcription factors of root and leaf gene regulatory networks. **Table S12.** List of TF-target interactions and nodes annotation of the leaf regulatory network. **Table S13**. Enriched GO terms for target genes of the TFs Myb (Solyc11g071300), TCP21 (Solyc03g006800) and EIL3 (Solyc01g006650). **Table S14.** List of predicted targets for SLIM1 (AT1G73730) TF and EIL3 (Solyc01g006650) according to PlantRegMap derived from literature, high-throughput assays and/or genome comparison, as well as the architectures and evolutionary features of plant transcription regulatory systems (http://plantregmap.cbi.pku.edu.cn/). **Table S15.** Differentially expressed *SULTR*, *APS* and *APR* genes by sulfate starvation in at least one sample (q-value < 0.05 and absolute log2FC > 1) in this work. **Table S16.** The primers used in qPCR analysis. (ZIP 17724 kb)

## Data Availability

The RNA-Seq datasets generated and analysed during the current study are available in the NCBI Sequence Read Archive (SRA) repository, accession PRJNA629977, available at https://www.ncbi.nlm.nih.gov/sra/PRJNA629977. All other data generated during this study are included in this published article and its supplementary information files.

## Abbreviations

S: Sulfur
PAPS: 3′-phosphoadenosine 5′-phosphosulfate
SULTRs: High affinity sulfate transporters
TFs: Transcription factors
EIL3: ETHYLENE-INSENSITIVE-LIKE3
SLIM1: SULFUR LIMITATION 1
GSLs: Glucosinolates
SD1: SULFUR DEFICIENCY 1
HY5: LONG HYPOCOTYL 5
MSA1: MORE SULPHUR ACCUMULATION 1
MS: Murashige and Skoog salts
LRT: Likelihood ratio test
TPM: Transcripts per million
FC: Fold Change
GO: Gene Ontology
FDR: False discovery rate
qPCR: Quantitative real-time PCR
ANOVA: Analysis of Variance
PCA: Principal Component Analysis
xyloglucan endotransglucosylase/hydrolase: XET
LSU: RESPONSE TO LOW SULFUR
EIL3: Ethylene Insensitive 3-Like3
