# Supplementary figures and images for: Transcriptomic analysis at organ and time scale reveals gene regulatory networks controlling the sulfate starvation response of Solanum lycopersicum
Source: BMC Plant Biol. 2020 Aug 24;20:385. doi: 10.1186/s12870-020-02590-2 (PMC7444261; doi:10.1186/s12870-020-02590-2)

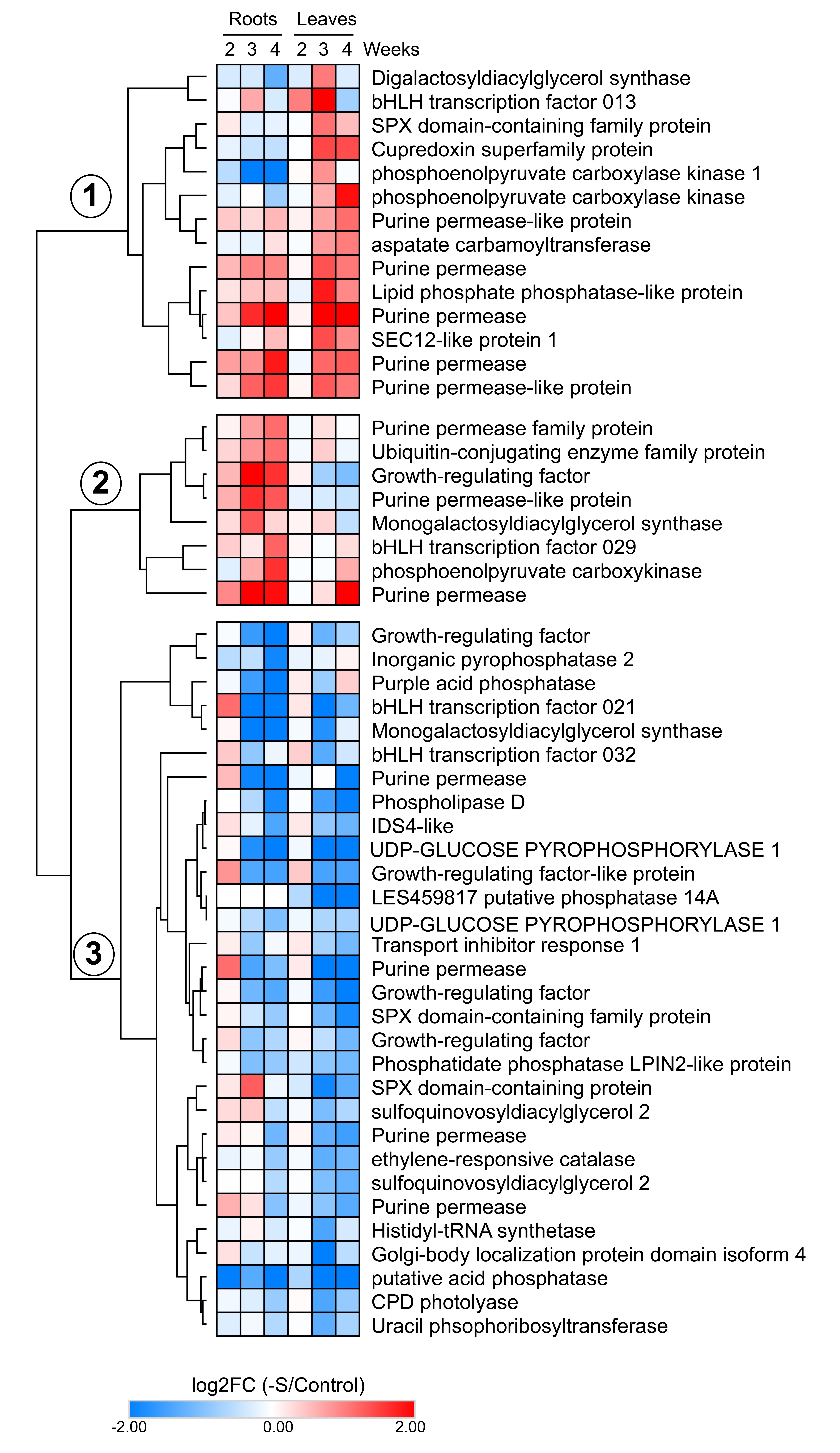

Supplement: Supplementary file 1 — Additional file 1: Figure S1. Total leaf area is reduced by sulfate deficiency from 3 weeks after sowing. Figure S2. A) Number of differentially expressed genes by sulfate starvation in roots, leaves or shared by both organs at 2, 3 and 4 weeks after sowing. B) Venn diagrams showing the overlap between early sulfate-regulated genes in roots and late regulated genes in leaves. Figure S3. qPCR validation of RNA-seq data. A) Correlation between relative mRNA levels of qPCR and RNA-seq data on selected transcripts. The list of selected differentially expressed genes by sulfate starvation is shown in Table S3. B) Heatmap comparing the expression patterns of selected genes analyzed by qPCR and RNA-seq. Figure S4. Hierarchical clustering of 52 genes included in the GO term “cellular response to phosphate starvation”, the most enriched GO term of sulfate-responsive orthologous gene families that are found exclusively in tomato plants. Figure S5. Co-expression clusters down-regulatedby sulfate starvation of genes significantly affected by sulfate and time in roots (left) and leaves (right) (q-value< 0.05 and absolute log2 FC > 1). On each box, the central mark indicates the median, and the bottom and top edges of the box indicate the 25th and 75th percentiles, respectively. Whisker indicates standard deviation of the expression data of all genes belongs to the cluster. Figure S6. Co-expression clusters up-regulated by sulfate starvation of genes significantly affected by sulfate and time in leaves (q-value< 0.05 and absolute log2 FC > 1). On each box, the central mark indicates the median, and the bottom and top edges of the box indicate the 25th and 75th percentiles, respectively. Whisker indicates standard deviation of the expression data of all genes belongs to the cluster. Figure S7. A) Distribution of Cluster 1 genes regulated by sulfate and time in leaves across different categories of the primary metabolism using Mapman4 annotation framework. B) Mapman analysis of sul [file 12870_2020_2590_MOESM1_ESM.zip › Figure S4.jpg]

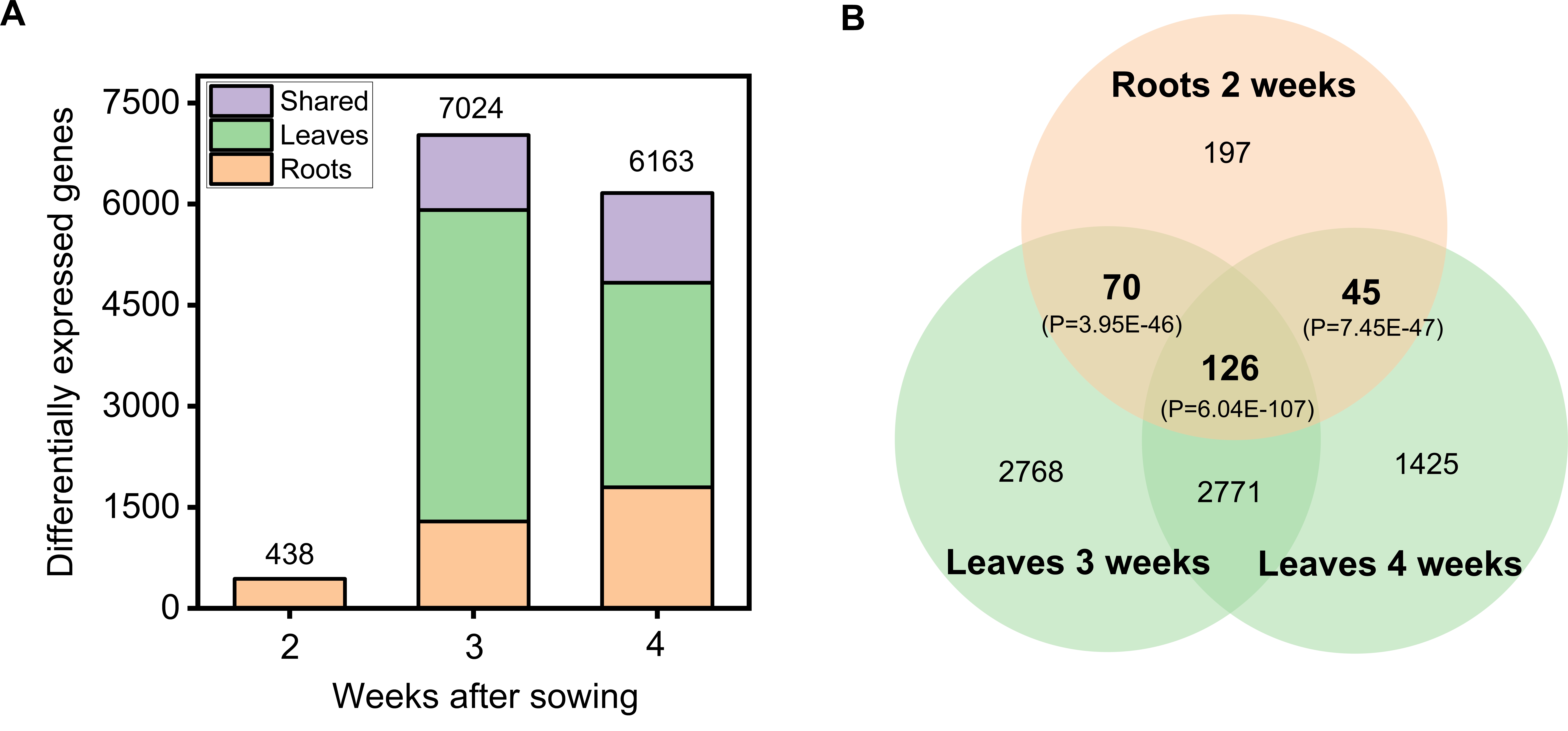

Supplement: Supplementary file 1 — Additional file 1: Figure S1. Total leaf area is reduced by sulfate deficiency from 3 weeks after sowing. Figure S2. A) Number of differentially expressed genes by sulfate starvation in roots, leaves or shared by both organs at 2, 3 and 4 weeks after sowing. B) Venn diagrams showing the overlap between early sulfate-regulated genes in roots and late regulated genes in leaves. Figure S3. qPCR validation of RNA-seq data. A) Correlation between relative mRNA levels of qPCR and RNA-seq data on selected transcripts. The list of selected differentially expressed genes by sulfate starvation is shown in Table S3. B) Heatmap comparing the expression patterns of selected genes analyzed by qPCR and RNA-seq. Figure S4. Hierarchical clustering of 52 genes included in the GO term “cellular response to phosphate starvation”, the most enriched GO term of sulfate-responsive orthologous gene families that are found exclusively in tomato plants. Figure S5. Co-expression clusters down-regulatedby sulfate starvation of genes significantly affected by sulfate and time in roots (left) and leaves (right) (q-value< 0.05 and absolute log2 FC > 1). On each box, the central mark indicates the median, and the bottom and top edges of the box indicate the 25th and 75th percentiles, respectively. Whisker indicates standard deviation of the expression data of all genes belongs to the cluster. Figure S6. Co-expression clusters up-regulated by sulfate starvation of genes significantly affected by sulfate and time in leaves (q-value< 0.05 and absolute log2 FC > 1). On each box, the central mark indicates the median, and the bottom and top edges of the box indicate the 25th and 75th percentiles, respectively. Whisker indicates standard deviation of the expression data of all genes belongs to the cluster. Figure S7. A) Distribution of Cluster 1 genes regulated by sulfate and time in leaves across different categories of the primary metabolism using Mapman4 annotation framework. B) Mapman analysis of sul [file 12870_2020_2590_MOESM1_ESM.zip › Figure S2.jpg]
